# Unmet need for contraception and its association with unintended pregnancy in Bangladesh

**DOI:** 10.1186/s12884-017-1379-4

**Published:** 2017-06-12

**Authors:** Ghose Bishwajit, Shangfeng Tang, Sanni Yaya, Zhanchun Feng

**Affiliations:** 1 School of Medicine and Health Management, Tongji Medical College, Huazhong University of Science and Technology, Wuhan, Hubei 430030 China; 2 School of International Development and Global Studies, University of Ottawa, Ottawa, ON K1N 6N5 Canada

**Keywords:** Unintended pregnancy, Unmet need for contraception, Reproductive health, Bangladesh

## Abstract

**Background:**

Unmet need for contraception and unintended pregnancy are important public health concerns both in developing and developed countries. Previous researches have attempted to study the factors that influence unintended pregnancy. However, the association between unmet need for contraception and unwanted pregnancy is not studied adequately. The aim of the present study was to measure the prevalence of unmet need for contraception and unwanted pregnancy, and to explore the association between these two in a nationally representative sample in Bangladesh**.**

**Methods:**

Data for the present study were collected from Bangladesh demographic and health survey conducted in 2011. Participants were 7338 mothers ageing between 13 and 49 years selected from both rural and urban residencies. Planning status of last pregnancy was the main outcome variable and unmet need for contraception was the explanatory variable of primary interest. Cross tabulation, chi-square tests and logistic regression (Generalised estimating equations) methods were used for data analysis.

**Results:**

Mean age of the sample population was 25.6 years (SD 6.4). Prevalence of unmet need for contraception was 13.5%, and about 30% of the women described their last pregnancy as unintended. In the adjusted model, the odds of unintended pregnancy were about 16 fold among women who reported facing unmet need for contraception compared to those who did not (95% CI = 11.63–23.79).

**Conclusion:**

National rates of unintended pregnancy and of unmet need for contraception remain considerably high and warrant increased policy attention. Findings suggests that programs targeting to reduce unmet need for contraception could contribute to a lower rate of unintended pregnancy in Bangladesh. More in-depth and qualitative studies on the underlying sociocultural causes of unmet need can help develop context specific solutions to unintended pregnancies.

## Background

Unintended pregnancy is regarded as a serious public health issue both in developed and developing countries, and has received growing research and policy attention during last few decades. According to Population Reference Bureau reports (2009 estimate), each year about 200 million cases of unintended pregnancies occur year worldwide [1]. Evidence suggests that a quarter of the unintended pregnancies end in unsafe abortions which is one of the leading causes of maternal mortality at the global stage [1]. In general terms, unintended pregnancy refers to pregnancies that are either mistimed (i.e. occurred at an unplanned or inopportune time), or unwanted (i.e. occurred when parents had no desire for more children) [2]. Unintended pregnancy has shown to result in a range of adverse physical and psychological health outcomes both for mother and infants [1, 3] affecting various socio-economic and cultural aspects of community health [4]. Evidence shows that unplanned/mistimed pregnancies are associated with higher rates of abortion and obstetric complications [5], poor utilisation of maternal healthcare services (MHS) [6], and postpartum depression and stress [7]. Apart from that, unintended pregnancies are associated with significantly increased risk of low birth weight (LBW) and preterm births (PTB) among children [8] and delayed and/or poor breastfeeding behaviour among mothers [9]. As a significant contributor to maternal and child morbidity and mortality, in resource poor countries especially the ones characterized by poor sexual and reproductive healthcare infrastructure, unintended pregnancy and abortion pose significant barriers to achieving the maternal and child health related Millennium Development Goals (MDGs) [10, 11].

With regard to MDG 4 and 5, Bangladesh has made impressive achievements especially in the indicators of family planning and contraceptive use, reduction in fertility rate, and reducing maternal and child mortality. Since 1990, maternal mortality rate (MMR) declined by 66% and infant mortality rate (IMR) by 57% [12]. During the period between 1975 and 2007, rate of utilisation of any type contraceptives among married women has increased sevenfold (8 to 56%) and that of modern methods has increased almost tenfold (5 to 48%) [13]. Rate of unintended pregnancy also declined in the country, however, at a slow pace from 33% in 1993 to 29% in 2011 [14]. Despite these achievements, the prevalence of unmet need for family planning still remains high and have been increasing albeit slowly in recent years. Demographic and Health Survey (DHS) defines unmet need for family planning as a situation of non-use of contraception when women are unwilling to have more children or want to have about two or more years later. According to Bangladesh Demographic and Health Survey (BDHS 2007), unmet need for family planning increased among both urban and rural women in all seven administrative divisions (11% in 2004 to 17% in 2007) [13]. One longitudinal study conducted on rural women in Bangladesh during 2006 and 2009 found that about a quarter of the women with unmet need for contraception experienced unwanted pregnancy [15]. These findings reveal that unmet need is a growing concern and can be regarded as a missed opportunity to address unintended pregnancy in the country.

The causes of unintended pregnancy are multifarious, however most commonly attributed to incorrect/non-use and discontinuation of contraceptives, contraceptive failure, unmet need for contraception [16, 17]. The concept of unmet need for contraception has a central position in the domain of family planning and reproductive health [15]. Globally, non-use of available contraceptives alone account for 90% of the unwanted pregnancies [12]. In developed countries, most abortions occur as a result of contraceptive failure and a small proportion are due to nonuse of contraception [17]. Contraceptive failure accounts for about 50% of all unintended pregnancies in the United States [18]. High rates of discontinuation and adoption of traditional contraceptive methods are major concerns for family planning programs in Bangladesh [13]. Addressing the challenges of and unmet need for contraception and unintended pregnancy should be a priority agenda for public health especially in countries like Bangladesh where abortion is not legal. Understanding the socioeconomic determinants of these issues are essential to develop effective strategies for the prevention of unintended pregnancies and provide services to the population in need of service. However, there remains a paucity of research evidence regarding the factors of association with unintended pregnancy in Bangladesh. In this study, we aim to evaluate the prevalence of unintended pregnancy and investigate the association between of unmet need for contraception and unwanted pregnancy. For this purpose, we used data from the latest demographic and health survey which is nationally representative and a reliable source of population health data in the country.

## Method

### About the survey and study population

The DHS survey program (www.measuredhs.com) has been operating in Bangladesh since 1993. The present study was based on data collected from the sixth wave of Bangladesh Demographic and Health Survey (BDHS) carried out in 2011. The survey is cross-sectional in nature and the sample population is nationally representative. The survey was conducted under the authority of the National Institute of Population Research and Training (NIPORT) and was implemented by Mitra and Associates, a well-known research institution in the country. Technical and financial assistance were provided by ICF International of Calverton (Maryland, USA) and United States Agency for International Development (USAID) respectively [13].

Bangladesh is the eighth most populous country in the world and third most populous in South Asia. It is also the most densely populated country in the world (excluding the city-states) with about 1015 inhabitants/km^2^ [13]. Due to rapid population growth, family planning policies to curb fertility rates have been in place throughout the country since its independence in 1971. The country is divided into seven administrative divisions (Barisal, Chittagong, Dhaka, Khulna, Rajshahi, Rangpur, and Sylhet) and survey was carried out in all these divisions encompassing both rural and urban population. The details of survey design, sampling method, data collection and distribution have already been described elsewhere [13, 19]. Briefly, the survey employed a two-stage stratified sampling of the households in a systematic way to ensure that the sample is nationally representative. In total 18,222 ever married women ageing between 12 and 49 years were selected for interview, of whom 17,842 were finally surveyed (response rate of 98%). The principle objectives of the survey were to provide most recent scenario of demographic (e.g. fertility, infant & maternal mortality rates), socioeconomic (e.g. literacy, employment, wealth status, food security), health indicators (e.g. malnutrition, rates of contraceptive use, skilled birth assistance, health literacy).

### Variable selection

The primary outcome variable of this study was pregnancy intention status among women for their last pregnancy. Though it is recognised among researchers that the concept of unintended pregnancy is a complex and nuanced one [17], it generally includes pregnancies described as mistimed or unwanted, and is usually used as a binary outcome (intended/unintended or wanted/unwanted). In this study it was measured by the response to the question on pregnancy intendedness and was dichotomized followingly: intended = wanted then, and unintended = wanted later/wanted no more.

Unmet need for contraception was the main exposure variable in this study. It was measured based on contraception utilisation status among participants who did/did not report desire for spacing or limiting childbirth. Unmet need for contraception refers to non-utilization of contraception measures among women who are fecund and sexually active, not want any more pregnancies or want to delay the next pregnancy. The concept of unmet need points to the gap between women’s reproductive intentions and their contraceptive behaviour. Participants who wanted to space/limit childbirth but not using contraception were considered as having unmet need for contraception.

For the selection of relevant covariates, we conducted a literature search in prominent medical databases for studies on unintended pregnancy. Based on literature review, and availability in the BDHS dataset, the following variables were selected for this study: age (<25/≥25); type of residence (urban/rural); educational attainment of respondent: nil (no formal education), primary (1–5 years of formal education); secondary/higher (>5 years of formal education); educational attainment of husband nil (no formal education); primary (1–5 years of formal education); secondary/higher (>5 years of formal education). husband’s occupation: blue collar (includes employments in farming, construction; white collar (includes employments in service, teaching, health sector, business); employment (yes/no); sex of household head (male/female); age at first birth (<18/≥18); ideal number of children (1/>1); has a say in own healthcare decision (yes/no); ever had a terminated pregnancy(yes/no); currently using any contraception (yes/no); unmet need for contraception(yes/no); decision maker for using contraception (yes/no); household wealth status (poor/middle/rich). DHS studies employs principal component analysis method to measure wealth index of households based on ownership of household assets e.g. durable goods (television, bicycle), household characteristics (sanitation facilities, construction materials) [13]. The factor scores are summed and standardized for each household which places them in a continuous scale based on relative wealth scores. Then the scores are categorized into quintiles where each households fall into a category, with the lowest scores representing the poorest and highest representing the richest households [19].

### Data analysis

The BDHS dataset contain information on wide range of variables. The dataset was checked to include only those participants for whom all information necessary for the present study were available. Prior to analysis, data were weighted by sample weights to generate population estimates. Descriptive analysis (frequency distribution) was performed to show the basic characteristics (e.g. demographic, socioeconomic, contraceptive use) of the sample population. Pearson’s *χ* ^2^ tests as well as cross tabulation were performed to show the group differences in terms of pregnancy intentions across the explanatory variables. At this stage, the explanatory variables were checked for multicollinearity to ensure validity for further analysis. Variables which showed statistically significant association (*p* < 0.05) in the *χ* ^2^ tests were included in the multivariable regression analysis. Given the complex clustered nature of the survey, we employed generalised estimating equation (GEE) method which serves a reliable tool for dealing with clustered data [19, 20]. GEE is a commonly employed statistical approach to fit a marginal model for clustered data analysis in clinical trials and biomedical studies [20, 21]. Adjusted odds ratios (OR) with 95% confidence intervals (CI) were calculated to measure the strength of associations between pregnancy intention status and the response variables in the model. A two-tailed *p*-value of <0.05 was used to assess the statistical significance for all tests. All analyses were carried out with the Mac Version of IBM SPSS Statistics 21.

## Results

Table 1 shows the basic characteristics (weighted) of the sample population. The mean age of the participants was 25.6 years (SD 6.4) and little less than half were aged below 25 years. More than a quarter of the women were of rural origin. Compared to husbands, women had a higher rate of literacy (80.7 vs 71.5%) and secondary/higher education enrollment rate (50.5 vs 42.5%). About one-fifth of the women had no formal education and half had secondary/higher education qualification. More than two-third of the husbands were engaged in blue collar activities and 27.3% in White collar profession. Well over one-third of the women reported low household wealth status and less than one-fifth of high wealth status. Majority of the women had no employment (89.8%) and were from male-headed households (92%). More than half of the women experienced first child-birth before reaching the age of 18 years. Almost all of the women (95.1) mentioned that single child is the ideal number of children for them. About one-third of the women reported having involvement in personal healthcare decision and little less than one-fifth ever terminating a pregnancy. About two-third of the women mentioned using any contraceptive method and 13.5% reported facing unmet need for contraception. Majority of the women (92.9%) reported making decisions for contraception by husband or jointly with husband and only 7.1% making decisions by themselves alone.

**Table 1:** Baseline Characteristics of the sample population. BDHS 2011

Variables	Operational definition	N (7338)	%
Age (25.6, SD 6.4)	Age of the participants at the time of the survey		
<25		3637	49.6
≥25		3701	50.4
Domicile	Location of residence of households		
Urban		1708	23.3
Rural		5630	76.7
Educational attainment	Total number of years of formal education		
Nil		1414	19.3
Primary		2216	30.2
Secondary/Higher		3708	50.5
Educational attainment of husband	Total number of years of formal education		
Nil		2092	28.5
Primary		2125	29.0
Secondary/Higher		3121	42.5
Husband’s occupation	Type of employment for income earning		
Blue collar		5336	72.7
White collar		2002	27.3
Wealth status	Availability of household materials		
Poor		3086	42.0
Middle		2901	39.5
Rich		1351	18.4
Employment	Working status of respondent at the time of survey		
Yes		750	10.2
No		6588	89.8
Sex of household head	Household head being a male figure		
Male		6754	92.0
Female		584	8.0
Age at first birth	Age at which respondent experienced first childbirth		
<18		3690	50.3
≥18		3648	49.7
Ideal number of children	Respondents opinion on the adequate number of children		
1		6979	95.1
>1		359	4.9
Has a say in own healthcare decision	Respondent can decide where to go and how much spending on healthcare		
Yes		4395	59.9
No		2942	40.1
Ever had a terminated pregnancy	History of pregnancy termination		
Yes		1338	18.2
No		6000	81.8
Currently using any contraception	Use of contraception of both traditional and modern type at the time of survey		
Yes		4836	65.9
No		2502	34.1
Unmet need for contraception	Fecund women who are not using contraception but want to space/limit pregnancy		
Yes		992	13.5
No		6346	86.5
Decision maker for using contraception	Decision on contraceptive use made by respondent alone or jointly with husband		
Respondent		520	7.1
Husband /Joint		6818	92.9

Results of chi-square tests presented in Table 2 are showing the factors of association with unwanted pregnancy. Cross-tabulation compares the relative percentage of participants reporting unwanted pregnancy against various demographic, socioeconomic and healthcare related factors. The rate of unwanted pregnancy was 29.3%. Results showed that the rate of unwanted pregnancy was higher among participants of rural origin, aging ≥25 years, higher educational status among both husband and wives and reporting poor wealth status. Women from male-headed households and had first childbirth before the age of 18 years were more likely to experience unwanted pregnancy. Women who reported the last pregnancy as desired were more likely have autonomy in own healthcare decision, using any contraception, had no unmet need for contraception, and had decision on contraception made by husband or jointly with husband.

**Table 2:** Percentage of participants reporting unintended pregnancy across the explanatory variables, BDHS 2011

Variables	Last pregnancy by intention status (*n* = 7338)		Chi-square	*p*-value
	Unintended (29.3)	Intended (70.7)		
Age				
< 25	40.0	53.5	111.62	<0.001
≥ 25	60.0	46.5		
Domicile				
Urban	21.6	24.0	4.74	0.031
Rural	78.4	76.0		
Educational attainment				
Nil	25.2	16.8	128.13	<0.001
Primary	34.1	28.6		
Secondary/Higher	40.8	54.6		
Educational attainment of husband				
Nil	33.7	26.4	53.36	<0.001
Primary	29.6	28.7		
Secondary/Higher	36.7	44.9		
Husband’s occupation				
Blue collar	76.3	71.2	20.22	<0.001
White collar	23.7	28.8		
Employment				
Yes	11.0	9.9	2.17	0.15
No	89.0	90.1		
Wealth status				
Poor	48.9	39.2	68.90	<0.001
Middle	36.8	40.7		
Rich	14.2	20.1		
Sex of household head				
Male	92.9	91.7	2.97	0.08
Female	7.1	8.3		
Age at first birth				
< 18	58.4	46.9	79.48	<0.001
≥ 18	41.6	53.1		
Ideal number of children				
1	94.8	95.2	0.584	0.441
> 1	5.2	4.8		
Has a say in own healthcare decision				
Yes	61.2	59.4	1.99	0.166
No	38.8	40.6		
Ever had a terminated pregnancy				
Yes	19.4	17.7	2.89	0.09
No	80.6	82.3		
Currently using any contraception				
Yes	71.9	63.4	371.23	<0.001
No	28.1	36.6		
Unmet need for contraception				
Yes	98.4	81.5	48.50	<0.001
No	1.6	18.5		
Decision maker for using contraception				
Respondent	9.9	5.9	35.83	<0.001
Husband /Joint	90.1	94.1		

### Results of multivariate analysis

Table 3 illustrates the factors associated with unwanted pregnancy in Bangladesh. Variables that did not show significant correlation in the chi-square bivariate test were removed from further analysis. Rest of the variables were entered in the regression model firstly singly (Model 1), secondly all at the same time (Model 2). In the univariate analysis (Model 1), compared to women who reported unmet need for contraception had significantly higher odds [OR = 13.13; 95%CI = 9.41–18.31] of experiencing unintended pregnancies compared to those did not report any. Unmet need for contraception appeared to be related with an increased likelihood of experiencing unintended pregnancy [OR = 16.24; 95%CI = 11.34–23.24)] after adjusting for age, type of domicile, educational attainment of respondent, educational attainment of husband, husband’s occupation, wealth status, sex of household head, age at first birth, ever terminating a pregnancy, currently using any contraception, decision maker for using contraception.

**Table 3:** Results of generalized estimating equations showing the factors associated with unwanted pregnancy in Bangladesh, BDHS 2011

	Model 1	Model 2
	COR (95% CI)	AOR (95% CI)
Unmet need for contraception (No)		
Yes	13.13 (9.41–18.31)	16.24 (11.34–23.24)

*Notes*: *AOR* Adjusted Odds Ratio, *COR* Crude Odds Ratio, *Model 1* Univariate analysis, *Model 2* Adjusted for age, type of domicile, educational attainment of respondent, educational attainment of husband, husband’s occupation, wealth status, sex of household head, age at first birth, ever terminating a pregnancy, currently using any contraception, decision making status for using contraception

## Discussion

### Main findings

Based on the analysis of Bangladesh Demographic and Health Survey 2011 data, this cross-sectional study reports the prevalence of unmet need for contraception and unintended pregnancy in a nationally representative population in Bangladesh. Findings of the analysis indicate a suboptimal utilization of contraception as about one-third of the eligible women were found to be non-users any contraceptive measures. Although about two-fifth of the women reported having the authority to make healthcare decisions on their own, only 7.1% said that they were the decision makers for contraception. The rate of unintended pregnancy seemed to have stagnated as there was practically no improvement compared to the 2004 demographic and health survey findings. However, the rate is still below the global average of 38% (2013 estimate) [22]. The rate of unmet need for contraception was 13.1%, which is slightly higher than the global average of 12.3% (2010 estimate) [23]. Women who reported richest wealth status were significantly less likely to report unmet need for contraception and unintended pregnancies compared to women from poorer households. Studies conducted in other countries have demonstrated that poverty is strongly correlated with both unmet need for contraception and unintended pregnancy [24]. Clearly, women from poorer households are less likely to be able to spend for personal healthcare such as reproductive health services and more likely to experience unintended pregnancies and other pregnancy related complications. Results of the multivariate analysis revealed a remarkably strong association between unmet need for contraception and unintended pregnancy. The association was statistically significant in both the unadjusted and adjusted model, however the level of association appeared to be stronger after adjusting for the potential confounders.

### Comparison with existing studies

In comparison with the findings from BDHS 2004, our result revealed that the rate of unintended pregnancy has remained virtually unchanged during this period (30% in 2004 vs 29.3% in 2011) [25, 26]. This rate is lower compared to the developing country average of 40% [27], but higher than neighbouring countries in South Asia [23, 28]. In line with previous evidences [29], our findings showed that both unmet need and non-use of contraception are positively associated with unintended pregnancy. However, unmet need showed the strongest association among all the predictive factors in the present study (Result not shown). Available evidence from previous studies suggests that non-use of whatever type (inadequate use of contraception, discontinuation, and contraceptive failure) is associated with increased likelihood of unintended pregnancy [30]. Moreover, as unintended pregnancy has been shown to be associated with increased risk of abortion and maternal morbidity [31], this remains a particular concern for women’s reproductive health in Bangladesh since induced abortion is not yet legalized and the scope of safe abortion is also limited, especially in the rural areas. In addition to unmet need, contraceptive discontinuation and non-use are also very common phenomenon in Bangladesh and contributes to unintended pregnancy [32].

#### General discussion

Unintended pregnancy is a global problem and has serious repercussions on child and maternal health. Many adolescents with unintended and unwanted pregnancies end up choosing abortion without knowing the associated risks and health consequences. Addressing the causes of unintended pregnancies will contribute to improved health status for mothers and their babies and help attain the maternal and child health related goals in the developing countries like in Bangladesh. Findings of the study supports the fact that programs addressing unmet need for contraception could result in lower prevalence of unintended pregnancies, which will consequently decrease the burden of unsafe abortion induced morbidities and mortalities. Therefore proper guidelines should be developed to measure the extent and underlying causes of unmet need in an effort to reduce the burden of unintended pregnancy in the country. Family programs aimed at reducing the rate of unmet need for contraception can prove highly beneficial for reducing the prevalence of unintended pregnancy in Bangladesh. Given the high rate of poverty and illiteracy, it is assumable that there is a greater risk of poor reproductive health outcomes due to inadequate knowledge on the risks and causes associated with contraception. More importantly, unmet need is a complex construct that may not be properly reflected through interview since it depends on individual perception of need for contraception and pregnancy.

#### Strength and limitations

This study has few notable limitations. Firstly, the survey is crosssectional, therefore cannot guarantee causal relationships. Secondly, unmet need and unintended pregnancy were measured based on self-reported data, which can be subject to recall error and degree of perception of the problem of participants. As the survey was conducted few years back, the prevalence of contraceptive use and unintended pregnancy might have changed across the population. Despite these limitations, our findings can provide useful insights for developing strategies to prevent unplanned conception in the country, and serves as a basis the future researches in this area.

## Conclusions

This study concludes that unmet need for contraceptives is strongly associated with unintended pregnancy among Bangladeshi women. The underlying socioeconomic factors that influence optimum utilization of contraceptives should be explored and addressed in order to achieve the fertility goals for the fast expanding population. Future researches along this line should focus on a broader range of sociocultural and behavioral factors that influence women’s perception and adoption of family planning and pregnancy.

## Abbreviations

DSH: Demographic and health survey
DSH: Family planning
GEE: Generalised estimating equation
ICDDR,b: International Centre for Diarrhoeal Disease Research, Bangladesh
MOHFW: Ministry of Health and Family Welfare
